# Evaluation of 2-deoxy-D-glucose as a chemotherapeutic agent: mechanism of cell death

**DOI:** 10.1038/sj.bjc.6600547

**Published:** 2002-09-23

**Authors:** R L Aft, F W Zhang, D Gius

**Affiliations:** Department of Surgery, Alvin J. Siteman Cancer Center, Washington University School of Medicine, St. Louis, Missouri, MO 63110, USA; John Cochran Veterans Administration Hospital, St. Louis, Missouri, MO 63110, USA; National Cancer Institute, Radiation Oncology Branch, National Institute of Health, Bethesda, Maryland, MD 20892, USA

**Keywords:** apoptosis, breast cancer, glucose metabolism, tumour metabolism

## Abstract

Nutrient deprivation has been shown to cause cancer cell death. To exploit nutrient deprivation as anti-cancer therapy, we investigated the effects of the anti-metabolite 2-deoxy-D-glucose on breast cancer cells *in vitro*. This compound has been shown to inhibit glucose metabolism. Treatment of human breast cancer cell lines with 2-deoxy-D-glucose results in cessation of cell growth in a dose dependent manner. Cell viability as measured by the 3-(4,5-dimethylthiazol-2-yl)-2,5-diphenyl tetrazolium bromide conversion assay and clonogenic survival are decreased with 2-deoxy-D-glucose treatment indicating that 2-deoxy-D-glucose causes breast cancer cell death. The cell death induced by 2-deoxy-D-glucose was found to be due to apoptosis as demonstrated by induction of caspase 3 activity and cleavage of poly (ADP-ribose) polymerase. Breast cancer cells treated with 2-deoxy-D-glucose express higher levels of Glut1 transporter protein as measured by Western blot analysis and have increased glucose uptake compared to non-treated breast cancer cells. From these results we conclude that 2-deoxy-D-glucose treatment causes death in human breast cancer cell lines by the activation of the apoptotic pathway. Our data suggest that breast cancer cells treated with 2-deoxy-D-glucose accelerate their own demise by initially expressing high levels of glucose transporter protein, which allows increased uptake of 2-deoxy-D-glucose, and subsequent induction of cell death. These data support the targeting of glucose metabolism as a site for chemotherapeutic intervention by agents such as 2-deoxy-D-glucose.

*British Journal of Cancer* (2002) **87**, 805–812. doi:10.1038/sj.bjc.6600547 www.bjcancer.com

© 2002 Cancer Research UK

## 

Tumour cells are dependent on glycolysis to support their metabolic requirements. Even under aerobic conditions, tumour cells continue to rely on glycolysis rather than oxidative phosphorylation (Warburg effect) resulting in high glucose requirements to generate energy and support metabolic function (Warburg, 1956; Weber, 1977). Malignant transformation of cultured cells with oncogenes or onco-viruses results in an absolute increase in the amount of glucose transported into the cell. This is mediated by transcriptional activation of the Glut1 glucose transporter gene resulting in increased levels of glucose transporter mRNA and protein (Birnbaum *et al*, 1987; Flier *et al*, 1987). Many human cancers have been found to express elevated levels of the Glut1 glucose transporter compared to normal tissues (Yamamoto *et al*, 1990; Younes *et al*, 1996). In human breast cancers, high Glut1 expression tends to correlate with tumours having high proliferative activity and histological score (Brown and Wahl, 1993; Younes *et al*, 1995). These findings have led to the hypothesis that increased glucose uptake may represent an important regulatory point in the maintenance of growth and synthetic activity of malignant cells and suppression of apoptosis (Greiner *et al*, 1994; Kan *et al*, 1994; Bentley *et al*, 2001; Vander Heiden *et al*, 2001).

Experimental conditions of glucose-deprivation have been demonstrated to cause apoptosis in many transformed cell lines (Shim *et al*, 1998). For example, glucose-deprivation in an interleukin 3 dependent lymphoma cell line and the MCF7/ADR breast cancer cell line have been found to cause apoptosis (Kan *et al*, 1994; Lee *et al*, 1997). Multiple related mechanisms may be involved in glucose-deprivation induced signalling including the activation of kinases (mitogen activation protein kinase, c-Jun N-terminal kinase, Lyn kinase) (Galoforo *et al*, 1996; Gupta *et al*, 1997; Liu *et al*, 1997; Lee *et al*, 1998), change in the redox state of the cell (Blackburn *et al*, 1999; Spitz *et al*, 2000), or generation of free radicals (Nomura *et al*, 1999).

Glucose-deprivation can be mimicked with glucose antagonists. Glucose analogues have been found to profoundly inhibit glucose metabolism in cancer cells *in vitro* and *in vivo* (Ball *et al*, 1957; Laszlo *et al*, 1958; Kaplan *et al*, 1990a). Of the many glucose analogues which have been investigated, 2-deoxy-D-glucose (2DG) has proved very effective in the inhibition of glucose metabolism and ATP production (Laszlo *et al*, 1960; Jain *et al*, 1985; Kaplan *et al*, 1990a). 2DG is a structural analogue of glucose differing at the second carbon atom by the substitution of hydrogen for a hydroxyl group (Figure 1A

**Figure 1:**
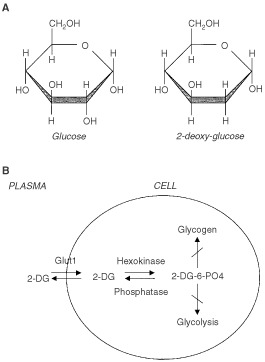
(**A**) Structural comparison of glucose and 2-deoxy-D-glucose. 2DG and glucose differ at the second carbon. (**B**) Schematic diagram of 2-DG action. 2DG enters the cell through the glucose transporter and is phosphorylated by hexokinase. Due to low levels of intracellular phosphatase, 2-DG-PO_4_ is trapped in the cell. 2-DG-PO_4_ is unable to undergo further metabolism. High intracellular levels of 2-DG-6-PO_4_ cause allosteric and competitive inhibition of hexokinase. This results in inhibition of glucose metabolism.

) and appears to selectively accumulate in cancer cells by metabolic trapping (Gallagher *et al*, 1978) due to increased uptake (Waki *et al*, 1998; Rivenzon-Segal *et al*, 2000), high intracellular levels of hexokinase or phosphorylating activity (Kaplan *et al*, 1990a; Arora *et al*, 1992; Waki *et al*, 1997; Aloj *et al*, 1999), and low intracellular levels of phosphatase (Kern and Norton, 1987; Nelson *et al*, 1996) (Figure 1B). Cell killing caused by 2DG has been described in several cell lines (Jain *et al*, 1985; Kaplan *et al*, 1990b; Nomura *et al*, 1999; Ko *et al*, 2001).

To exploit glucose metabolism of cancer cells as a therapeutic target, we have investigated the effects of 2DG in human breast cancer cells. In the described series of experiments, we demonstrate that 2DG inhibits breast cancer cell growth, clonogenicity, and causes cell death through apoptosis. Breast cancer cells treated with 2DG express higher levels of the Glut1 transporter protein and exhibit increased uptake of glucose, indicating that cells might enhance their death in response to 2DG treatment. These results support targeting glucose metabolism as a target site for chemotherapeutic intervention using compounds such as 2DG since the initial response of increased transporter expression and glucose uptake results in accelerated cellular demise.

## MATERIALS AND METHODS

### Cell culture

Cell lines SkBr3, MCF-7, BT474, and MDA/MB468, all obtained from ATCC, were grown in RPMI-1640 media supplemented with 10% heat inactivated fetal calf serum, penicillin (100 units ml^−1^) streptomycin (100 μg ml^−1^), and amphotericin B (0.25 μg ml^−1^) in a humidified atmosphere of 5% CO_2_ and 95% air at 37°C. For most experiments, cells were seeded at 2.5×10^6^ cells per 10-cm diameter tissue culture plate 2 days prior to use.

### Clonogenic survival assay

Cells were plated at 4000 cells well^−1^ in a 96-well plate, allowed 24 h to attach and subsequently exposed for 4 h to 2DG. Cells were trypsinised, counted, and replated at 400 cells per 60 mm-diameter culture dish and incubated undisturbed for 14 days at 37°C in a humidified incubator. Colonies were counted after fixing with methanol: acetic acid (3:1) and staining with 0.25% crystal violet. Colonies containing more than 25 cells were scored as positive. Each data point was performed in triplicate. Data were normalised to sham treated control cell plating efficiencies. Plating efficiency of sham treated cells was 38–50%, calculated as the number of colonies observed divided by the number of trypsinised cells plated. Error bars represent the range of values. Each data point represents the average of three samples.

### Measurement of [^14^C]3-O-methyl-glucose (3-O-MG) uptake

One to two days prior to the experiment, cells were plated in triplicate at 10^4^ cells cm^−2^ in 6-well plates (35 mm diameter wells). Cells were incubated with 8 mM 2DG for 4 h, rinsed with warm PBS, and 950 μl of warm uptake media (128 mM NaCl, 4.7 mM KCl, 1.25 mM MgSO_4_, 10 mM NaH_2_PO_4_, 1.25 mM CaCl_2_, pH 7.4) was added to each well. Where indicated, 50 μM of cytochalasin B was added in the warm uptake media (Hamrahian *et al*, 1999). Plates were incubated at 37°C for 6 min and subsequently 50 μl of [^3^H]3-O-methyl-glucose was added (1 mM 3-O-methyl-glucose, 20 μCi ml^−1^ of [^14^C]3-O-methyl-glucose). Incubation was continued for the indicated times. The reactions were stopped by the addition of cold PBS. The cells were washed five times with cold PBS and lysed with 1 mM NaOH. Ten microlitres from each sample was used for protein concentration analysis (Bio-Rad DC Protein Assay). Scintillation fluid was added to the remainder of the sample for scintillation counting. Glucose uptake was calculated as μmoles uptake per μg protein. 3-O-methyl-D-[^14^C]glucose (56 mCi mmol^−1^) was purchased from Amersham Corp.

### MTT assay

Cells (2500 per well) were plated in quadruplicate in 96-well culture plates. Following cell adherence (24–48 h), experimental media containing 2DG or control media was added and incubation continued for the indicated times at 37°C. MTT (0.5 mg ml^−1^ in PBS) was added to each well and incubation continued at 37°C for an additional 4 h. The media was then discarded and 200 μl of DMSO was added to each well to solubilise the coloured formazan product. Absorbance was read at 550 nm on a scanning microtiter spectrophotometer plate reader (Bio-Rad) after agitating the plated for 5 min on a shaker. All data is expressed relative to untreated cells in the same experiment which are standardised to 100% (Carmichael *et al*, 1987).

### Glut1 protein Western blot analysis

Cells were plated at 10^4^ cells cm^−2^ and grown in the presence of 2DG for the times indicated in the figures. At each time point, cells were collected, and lysed by the addition of HER buffer (250 mM sucrose, 10 mM HEPES, 1 mM EDTA (pH 8.0)). DNA was fragmented by passing the solution through a 21g needle 3–4 times. Protein concentration was determined using the Bio-Rad DC Protein Assay. Samples were mixed with 2×Laemmli lysis buffer (1×=2.4 M glycerol, 0.14 M Tris, pH 6.8, 0.21 M sodium dodecylsulphate, 1.28 M mercaptoethanol, 0.3 mM bromophenol blue). Protein (50 μg) was added per lane on a 10% separation SDS-polyacrylamide gel (acrylamide:bis-acrylamide 20:1). Gels were run at 200 V in 25 mM Tris, 192 mM Glycine, 0.1% SDS and resolved proteins were transferred to nitrocellulose overnight at 4°C 22 V in 25 mM Tris, 192 mM Glycine, 20% v v^−1^ methanol buffer. To block non-specific sites, filters were incubated with Blotto (5% nonfat dry milk, 0.02% sodium azide, 0.01% Antifoam A in PBS) for 1 h at room temperature. Blots were probed with anti-Glut1 polyclonal antibody by incubation at room temperature for 1 h. This antibody was generated against the C-terminal 13 amino acids (gift from Dr. Mike Meckler) diluted 1 : 1000, or purchased from DAKO (Marshall *et al*, 1993). The membrane was washed with PBS and subsequently incubated with the secondary antibody horseradish peroxidase conjugated donkey anti-rabbit IgG (diluted 1 : 500) for 1–2 h. The membrane was rinsed in Blotto, and the amount of binding visualised by using enhanced chemiluminescence method (ECL, Amersham Pharmacia Biotech). Film exposure ranged from 10 s to 1 min. The blots were stained with 0.5% Ponceau in 5% TCA to confirm uniform protein transfer prior to antibody reactions. Where indicated the membranes were stripped in 100 mM β-mercaptoethanol, 2% SDS, 62.5 mM Tris-HCl pH 6.8 at 55°C for 30 min and re-probed with anti-actin antibody (diluted 1 : 40 obtained from Calbiochem) for 1 h at room temperature. Secondary antibody incubation and development were performed as described.

### PARP Western blot analysis

Cells were grown as above and lysed with buffer containing 62.5 mM Tris pH 6.8, 8 M deionised urea, 10% glycerol, 2% SDS and protease inhibitor stock solutions (final concentration: 1 μg ml^−1^ Leupeptin, 1 μg ml^−1^ antipain, 1 μg ml^−1^ benzamidine, trypsin inhibitor 5 μg ml^−1^, 1 μg ml^−1^ chymostatin, 1 μg ml^−1^ pepstatin A, 87 μg ml^−1^ PMSF). Samples were sonicated for 20 s to fragment the DNA. Samples containing 50 μg of protein were heated to 65°C and size-separated protein on 7.5% gel. Protein was transferred to nitrocellulose as described above. After blocking non-specific binding sites with Blotto, filters were probed with anti-PARP antibody diluted 1 : 5000 (Biomol) for 2.5 h at room temperature. Filters were washed with PBS and subsequently incubated with the secondary antibody anti-mouse Ig conjugated to horseradish peroxidase diluted 1 : 2000 for 1 h at room temperature. Antibody binding was determined using ECL as described above. Positive controls were SkBr3 cells treated with 2 μM staurosporine for 6 h.

### Caspase-3 assay

Caspase 3 activity was determined using ApoAlert Caspase-3 Assay Kit (Clontech). 2×10^6^ cells were used for each time point. After the indicated treatments, cells were lysed by the addition of 50 μl of the provided lysis buffer and incubation on ice for 10 min. The non-soluble debris was removed by centrifugation at 14 000 r.p.m. for 3 min. Fifty microlitres of the supernatant was mixed with 50 μl of the provided reaction buffer. Where indicated, 0.5–1 μl of 1 mM DEVD-Fmk inhibitor was preincubated with the indicated specimens at 37°C for 30 min. Five microlitres of substrate 1 mM DEVD-pNA was added to each sample and the reaction allowed to run at 37°C for 1 h. Samples incubated without substrate served as the negative control. Absorbance was measured at 405 nm and using 655 nm as an internal reference.

## RESULTS

### Effect of 2DG on cell growth, clonogenic survival, and viability

**Figure 2:**
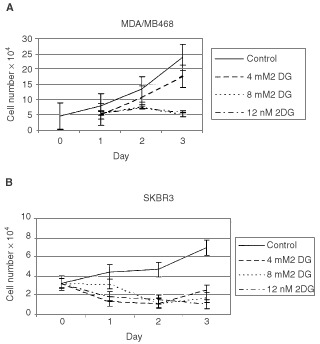
Effect of 2-DG on growth of breast cancer cell lines. SkBr3 and MDA/MB468 breast cancer cell lines were grown in the absence or presence of various concentrations of 2DG. 2DG was added on day zero and the media was not changed during the duration of the experiment. Cell number was determined daily. Each point represents triplicate cultures.

Glucose-deprivation has been shown to cause apoptosis in breast cancer cell lines (Lee *et al*, 1997). To determine whether a similar result could be achieved using the non-metabolisable glucose analogue 2DG, which is known to inhibit glucose metabolism (Wick *et al*, 1957), four human breast cancer cell lines MCF-7, SkBr3, BT549, and MDA/MB468 were grown in the presence of varying concentrations of 2DG. Representative growth curves from two of these cell lines are shown in Figure 2

. 2DG was added to cells on day zero at concentrations ranging from 4–12 mM. As seen in Figure 2A, growth of MDA/MB468 cells was completely inhibited by treatment with 8 mM 2DG. Treatment with 4 mM 2DG resulted in approximately 30% inhibition of cell growth. Of the breast cancer cell lines tested, SkBr3 was the most sensitive to the growth inhibitory effects of 2DG. Cell growth was completely inhibited at 4 mM 2DG (Figure 2B). This represents a ratio of 2DG to glucose in the culture media of 1 : 4.25. This is consistent with previous cell culture studies in which a ratio of 1 part 2DG to 3 parts glucose inhibited glycolysis approximately 50% (Laszlo *et al*, 1960). These experiments indicate that 2DG inhibits breast cancer cell growth which may be due to either growth arrest or cell death and that competition for essential sites of inhibition is in favour of 2DG.

**Figure 3:**
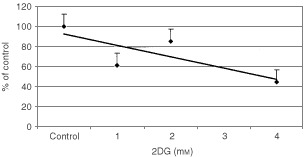
Clonogenic survival after 2DG treatment. SkBr3 cells were treated with varying concentrations of 2DG for 4 h and replated at low density. Colonies were counted 14 days after plating and those containing 25 cells or greater were scored as positive. Each point represents triplicate cultures.

It has previously been reported that normal cells undergo withdrawal from the cell cycle during nutrient or glucose deprivation rather than undergo cell death (Shim *et al*, 1998). To determine whether treatment with 2DG resulted in cell death or withdrawal from the cell cycle, clonogenic survival and MTT assays were performed. For the clonogenic survival experiments, SkBr3 cells were treated with 2DG for 4 h, re-cultured at low density in normal media, and colony formation determined at 14 days. As seen in Figure 3

, there is a dose dependent decrease in clonogenic survival with 2DG treatment. Treatment with 4 mM 2DG for 4 h results in 50% decrease in cell survival at 14 days compared to non-treated control cells. These results indicate that 2DG treatment causes cell death as reflected by decreased clonogenicity rather than withdrawal from the cell cycle.

**Figure 4:**
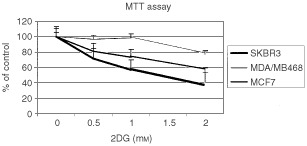
MTT conversion after 2DG treatment. SkBr3, MDA/MB468, and MCF7 cells were incubated with the indicated concentrations of 2DG for 4 h. MTT was added the amount of reduced formazan product determined spectrophotometrically. Results are expressed as per cent of control non-treated cells.

MTT is reduced by viable cells to a coloured formazan product and has been previously found to correlate with clonogenic survival and has been used for chemosensitivity testing (Carmichael *et al*, 1987). To determine whether reduction of MTT paralleled clonogenic survival, breast cancer cell lines were treated with varying concentrations of 2DG and spectrophotometric conversion of MTT measured. As seen in Figure 4

, 2DG treatment of three breast cancer cell lines for 4 h resulted in a dose dependent decrease in absorption at 550 nm indicting decreased reduction of MTT to its formazan product. Treatment with 1 mM 2DG resulted in an approximately 40% decrease in the reduction of MTT by SkBr3 cells, approximately 25% decrease by MCF7 cells, and no change by the MDA/MB468 cell line. When SkBr3 cells treated with 3 mM 2DG for 24 h and allowed to recover for 6 h a slight increase in MTT reduction is observed (50% *vs* 61%, data not shown). As observed in the cell growth experiments, the SkBr3 cell line appeared to be more sensitive to the effects of 2DG than MCF-7 and MBA/MB468 breast cancer cell lines in this assay. In addition, these results parallel the results of the clonogenic survival assay and indicate that the MTT conversion correlates with cell survival with 2DG treated cells.

### Caspase 3 activity and PARP cleavage with 2DG treatment

**Figure 5:**
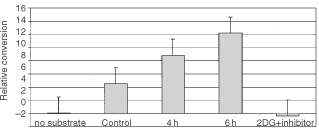
Caspase 3 activation in SkBr3 cells treated with 2DG. SkBr3 cells were treated with 16 mM 2DG for the 4 or 6 h. Cells were harvested and caspase 3 activity measured using a spectrophotometric assay according to the manufacturer's instructions. The caspase 3 inhibitor DEVD-FMK was added 30 min prior to the addition of substrate in cells which had been incubated with 16 mM 2DG for 6 h. Each point represents the average of triplicate cultures. Control cells were incubated for 6 h in the presence of media only.

Clonogenic survival and MTT assays encompass all types of cell death including necrosis and apoptosis. Apoptotic cell death is an active death process and can be inferred by measuring the activation of caspases and the cleavage of key enzymes. To determine whether the cell death caused by 2DG involves apoptosis, cells were examined for caspase 3 activity and PARP cleavage. Caspase 3 is an essential protease activated during apoptotic cascade (Gross *et al*, 1999). Caspase 3 activity was measured in SkBr3 cells after treatment with 2DG using a spectrophotometric assay based on the cleavage of DEVD-pNA, a substrate for caspase 3. As seen in Figure 5

, treatment of SkBr3 cells with 2DG results in a time dependent increase of caspase 3 activity. Caspase 3 activity increased by three-fold over control cells after treatment with 16 mM 2DG for 6 h. This activity was inhibited by pre-incubation with the caspase 3 inhibitor DEVD-FMK.

**Figure 6:**
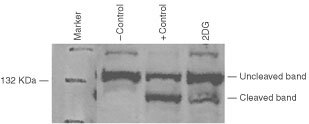
PARP activation by SkBr3 cells treated with 2-DG. Cells were treated for 6 h with 12 mM 2DG or 2 μM staurosporine (+control). Western blot analysis was performed using a monoclonal antibody to PARP. Normal (uncleaved) and the PARP cleavage product are indicated.

Poly (ADP-ribose) polymerase (PARP) is involved in DNA cleavage during apoptosis. During apoptosis the 116 kDa PARP enzyme is cleaved by caspases to an 89 kDa form which is no longer stimulated by nicked DNA (Shah *et al*, 1995). PARP cleavage was determined after treatment of SkBr3 cells with 2DG. In this experiment, SkBr3 cells were treated with either 12 mM 2DG for 6 h or 2 μM staurosporine. Staurosporine has previously been shown to be a potent inducer of apoptosis and was used as a positive control (Tafani *et al*, 2001). Cleavage of PARP was determined by Western blot analysis using a monoclonal antibody to PARP. As seen in Figure 6

, control cells had undetectable amounts of the PARP cleavage product (lane 1). In contrast, SkBr3 cells with 2DG or staurosporine resulted in the appearance of the 89kDa PARP cleavage product. Cells treated with 2DG had approximately 20% cleavage of the protein (lane 3). In addition, 2DG treatment of SkBr3 cells leads to DNA cleavage (unpublished data). These results together with the results of the caspase 3 activity experiments indicate that cell death caused by 2DG in SkBr3 cells occurs in part through apoptosis.

### Glut1 transporter expression and glucose uptake with 2DG treatment

**Figure 7:**
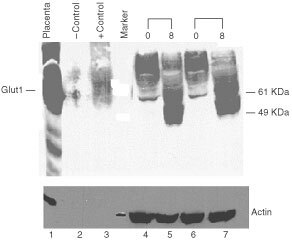
Glut-1 transporter protein levels in breast cancer cells lines treated with 2-DG. SkBr3 breast cancer cells were treated with 2DG for 48 or 72 h with 8 mM 2DG (8) or without 2DG (0). Protein was isolated, size separated on a 10% polyacrylamide gel. A Western blot was performed using a polyclonal anti-Glut-1 antibody. Positive controls are protein isolated from human placenta and membranes isolated from Glut1 injected *Xenopus* oocytes (+control). Sham injected oocytes were used as a negative control (−control). Glut1 protein is indicated. Molecular weight markers are labelled. The protein blot was reacted with anti-actin antibody after removal of the Glut1 antibody (lower panel).

The Glut1 glucose transporter is an integral membrane glycoprotein and is responsible for constitutive glucose uptake in breast cancer cells. Glut1 protein expression is increased in cancer cells and has been reported to increase during cellular stress and upon glucose deprivation (Kitzman *et al*, 1993; Blackburn *et al*, 1999). Glut1 contains an N-glycosylation consensus sequence in the first exofacial loop. As a result of heterogeneous glycosylation at this site, the transporter migrates as a broad band on SDS–PAGE gels (Marshall *et al*, 1993). Previous studies have demonstrated that glucose deprivation alters the glycosylation pattern of Glut1 resulting in a lower molecular weight species of the transporter which migrates as an approximately 37 kDa protein (Kitzman *et al*, 1993). This transporter species undergoes appropriate trafficking to the plasma membrane (Macmahon and Frost, 1995; Macmahon *et al*, 2000). To determine the effect of 2DG on Glut1 transporter levels and glycosylation pattern, SkBr3 cells were harvested after treatment with 8 mM 2DG for 48 or 72 h, and Glut1 expression determined by Western blot analysis using a rabbit polyclonal antibody prepared to a 13 amino acid C-terminal fragment of Glut1 (Marshall *et al*, 1993). Fully glycosylated Glut1 migrates as a broad band at approximately 47 kDa. This can be seen in Figure 7

, lanes 1 and 3. Control lysates demonstrating high Glut1 transporter expression are protein isolated from human placenta (lane 1) and plasma membranes isolated from *Xenopus* oocytes injected with a human Glut1 expression vector (lane 3) (Carayannopoulos *et al*, 2000). Lane 2 represents protein isolated from sham injected *Xenopus* oocytes. SkBr3 cells were incubated with either 0 or 8 mM 2DG for 48 or 72 h and protein extracts prepared. As seen in lanes 5 and 7, 2DG treatment results in the appearance of a lower molecular weight glycoform of Glut1 is seen which migrates as a 37-kDa protein. This is in contrast to protein isolated from cells which were not incubated with 2DG which had very low levels of Glut 1 protein (Figure 7, lanes 4 and 6). In addition, there is greater intensity of the bands reflecting higher levels of Glut1 protein. The same blot probed with anti-actin antibody demonstrated similar amounts of actin in each sample. This experiment indicates that 2DG treatment of SkBr3 cells results in increased amounts of Glut1 protein as well as a lower molecular weight protein which is likely the result of an altered glycosylation pattern.

**Figure 8:**
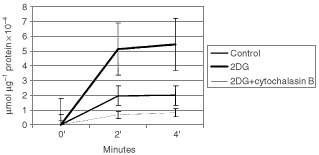
Glucose uptake by SkBr3 cells treated with 2-DG. SkBr3 cells were treated with 8 mM 2-DG and [^14^C]3-O-methyl-glucose uptake measured. Cytochalasin B (50 μM) was added simultaneously with the radioactive glucose.

To determine whether glucose uptake increased or decreased with 2DG treatment, glucose uptake was measured in SkBr3 cells after treatment with 8 mM 2DG for 4 h using the non-metabolisable glucose analogue 3-O-methyl-glucose (3-O-MG). 3-O-MG is not appreciably phosphorylated by cells and reaches equilibrium between extracellular and intracellular spaces. Specific uptake of 3-O-MG by the Glut1 transporter is inhibited by cytochalasin B (Hamrahian *et al*, 1999). As shown in Figure 8

, 3-O-MG uptake is three-fold higher in 2DG treated cells than control cells. Increased uptake of 3-O-MG observed with 2DG treatment was inhibited by pretreatment with cytochalasin B. This data indicates that 2DG treatment of breast cancer cells results in increased glucose uptake which may be due to higher levels of Glut1 protein.

## DISCUSSION

Breast cancers exhibit an increased rate of glucose uptake and express high levels of glucose transporter protein (Wahl *et al*, 1991; Brown and Wahl, 1993; Younes *et al*, 1995). Recently, glucose-deprivation has been reported to cause apoptosis in breast cancer cells (Lee *et al*, 1997). These observations lead us to hypothesise that interference with glucose metabolism by non-metabolisable glucose analogues would lead to apoptosis in breast cancer cells. Previous studies have demonstrated that interference with glycolysis by the non-metabolisable glucose analogue 2DG can cause cell killing through interference with glycolysis (Kaplan *et al*, 1990a; Nomura *et al*, 1999; Ko *et al*, 2001). The experimental results presented here extend previous observations and confirm our hypothesis by demonstrating that glucose-deprivation induced by 2DG leads to cessation of cell growth, decreases clonogenicity, and causes apoptosis in breast cancer cell lines. Furthermore, breast cancer cells treated with 2DG express higher levels of Glut1 transporter and demonstrate increased glucose uptake as a result of oxidative stress. Therefore, 2DG appears to be an effective agent for causing breast cancer cell death due to the initial response of accelerated glucose uptake by the stressed cells.

It has been hypothesised that increased glucose uptake may represent an important regulatory point in maintaining the growth and synthetic activity of malignant cells and suppression of apoptosis (Greiner *et al*, 1994; Kan *et al*, 1994). For example, c-Myc transformed rat fibroblasts up-regulate glucose transport and glycolytic gene expression through trans-activation (Osthus *et al*, 2000). These cells will undergo apoptosis upon withdrawal of glucose (Shim *et al*, 1998). In normal cells, growth is regulated by external growth signals and nutrient support (Rathmell *et al*, 2000; Vander Heiden *et al*, 2001). Cancer cells, in contrast, have lost responsiveness to most external growth signalling and as a consequence, nutrient supply in the form of glucose likely plays a unique role in maintaining cancer cell viability. Thus, normal and transformed cells respond to nutrient depletion or glucose-deprivation in opposing manners. Whereas normal cells compensate by increased glucose transporter expression or modification, transformed cells are stressed by glucose-deprivation leading to the expression of an array of stress related genes, which is subsequently followed by cell death. In many normal cell types, glucose deprivation results in an increase in the maximum velocity of glucose transport (Kitzman *et al*, 1993). This has been attributed to one of several mechanisms; translocation of transporter from an intracellular compartment to the plasma membrane (Haspel *et al*, 1991), changes in the glycosylation pattern of the Glut1 transporter with decreased turnover of the protein (Macmahon and Frost, 1995), or by increased synthesis of m RNA and protein (Reed *et al*, 1990; von der Crone *et al*, 2000). For example, pre-adipocyte cell lines respond to glucose starvation by increasing transporter expression reflected in elevated levels of Glut1 transporter mRNA and protein (Reed *et al*, 1990). 3T3 cells respond to glucose-deprivation by conversion of the native Glut1 transporter to an incompletely glycosylated form, while increasing basal transport 4.5-fold (Reed *et al*, 1990; Macmahon and Frost, 1995). Prolonged glucose deprivation, in non-transformed cell lines, causes cell-cycle arrest in G_0_/G_1_, which is reversible (Shim *et al*, 1998). In contrast, many transformed cell lines respond to glucose deprivation initially by expression of an array of stress related genes and subsequently undergoing apoptosis (Lee and Corry, 1998). For example, early in glucose deprivation, MCF7/Adr cells have increased levels of Lyn and c-Jun kinases as well as increased expression of bFGF and c-Myc mRNAs (Galoforo *et al*, 1996; Gupta *et al*, 1997; Liu *et al*, 1997; Lee *et al*, 1998). These results support the hypothesis that cancer cells are uniquely sensitive to glucose-deprivation and glucose-deprivation results in cellular stress and subsequent cytotoxicity.

Glucose-deprivation can be mimicked with glucose antagonists. Glucose analogues have been found to profoundly inhibit glucose metabolism in cancer cells *in vitro* as well as *in vivo* (Ball *et al*, 1957; Laszlo *et al*, 1960). Of the many glucose analogues, which have been investigated, 2DG appears to be the most effective in inhibiting glycolysis (Laszlo *et al*, 1958). 2DG is a structural analogue of glucose differing at the second carbon atom by the substitution of hydrogen for a hydroxyl group and appears to selectively accumulate in cancer cells (Figure 1). 2DG undergoes facilitated diffusion into cells via glucose transporters. Once intracellular, 2DG is phosphorylated by hexokinase to 2-deoxy-D-glucose-6-phosphate (2-DG-6-P). 2-DG-6-P is not a substrate for glucose-6-phosphate dehydrogenase or phosphohexoisomerase (Wick *et al*, 1957). Therefore, once formed, 2-DG-6-P is not further metabolised and will accumulate in the cell until de-phosphorylated by phosphorylase (Gallagher *et al*, 1978; Kern and Norton, 1987; Nelson *et al*, 1996). In malignant cells, specific accumulation of 2DG is attributed to several mechanisms; increased glucose transporter expression resulting in increased uptake (Waki *et al*, 1998), increased hexokinase activity or phosphorylating activity resulting in trapping (Kaplan *et al*, 1990a; Aloj *et al*, 1999), and low phosphorylase activity resulting in accumulation (Kern and Norton, 1987; Nelson *et al*, 1996). Inhibition of glycolysis by 2-DG is thought to occur by inhibition of key glycolytic enzymes such as phosphohexoisomerase (Horton *et al*, 1982) as well as depletion of high energy compounds. 2-DG-6-P may act as a potent P_i_ trap resulting in depletion of intracellular stores. Low P_i_ rapidly leads to dephosphorylation of ATP with a concomitant increase in AMP. High AMP and low P_i_ stimulates AMP deaminase, which converts AMP to inosine and leads to a decrease in the total adenine pool. The low P_i_ and ATP makes hexokinase far more susceptible to inhibition by G-6-P which may occur by feedback inhibition and/or allosteric inhibition (Ardehali *et al*, 1996, 1999).

Hexokinase may be the key regulator in 2DG-induced apoptosis. Hexokinase is the first enzyme of the glycolytic reaction Glucose+MgATP ->Glucose-6-PO_4_ + MgADP which commits glucose to undergo metabolism. The phosphorylation of glucose by hexokinase ensures glucose entry into cells via facilitated diffusion along a downhill concentration gradient. Four isoenzymes of hexokinase are found in mammalian tissues and are subject to allosteric inhibition by the product Glucose-6-PO_4_ (Wilson, 1997). Type II hexokinase, the isoform over-expressed in transformed cells, has the highest Km for glucose (Parry and Pedersen, 1983; Rempel *et al*, 1996). Markedly elevated levels of hexokinase II have been described in many malignant cell lines and unlike normal tissue, 50–80% of the enzyme is bound to the mitochondrial fraction, where it is thought to have preferential access to intramitochondrially generated ATP and be more resistant to Glucose-6-PO_4_ inhibition (Arora and Pedersen, 1988; Arora *et al*, 1992). In addition, hexokinase forms one component of the proposed permeability transition pore, which is involved in mitochondria depolarisation following apoptotic signals (Gross *et al*, 1999). Recently published data supports a role of hexokinase activity in the prevention of apoptosis mediated by Akt (Gottlob *et al*, 2001). Thus, it is possible that hexokinase expressed in cancer cells may be uniquely sensitive to 2DG which may have a direct effect on the apoptotic signal pathway through its role as a member of the permeability transition pore complex.

A related mechanism for the triggering of apoptosis by glucose-deprivation or 2DG may be induction of stress-related signals caused by an imbalance in the energy state of the cell subsequent to ATP depletion. One method to avoid apoptosis would be to maintain a favourable energy balance. Should the latter fall below a certain crucial point, the pre-existing program for apoptosis would be executed through the expression of stress related genes (Kan *et al*, 1994). Previous studies have shown that 2DG inhibits the rate of glucose usage and glycolysis cancer cell lines by 15–40% resulting in reduced ATP levels by nearly 40% tested cell lines (Kaplan *et al*, 1991; Dwarkanath *et al*, 2001). Glucose-deprivation results in activation of the MAP kinase pathway as a result of oxidative stress (Lee *et al*, 1998; Blackburn *et al*, 1999; Spitz *et al*, 2000). This pathway is suppressed by over-expression of Bcl-2 which is reported to suppress apoptogenic activity involving mitochondria through an antioxidant pathway (Hockenbery *et al*, 1993; Zhong *et al*, 1993; Lee *et al*, 1997; Lee and Corry, 1998; Shim *et al*, 1998). This would support a role for alteration in the energy balance of the cell in induction of apoptosis with glucose deprivation or 2DG treatment.

In this report we present data demonstrating that 2DG treatment of breast cancer cells leads to cell death which occurs in part by apoptosis. Four breast cancer cell lines were tested which demonstrated varying sensitivity to the cytotoxic effect of 2DG. SkBr3 cells were the most sensitive to the effects of 2DG demonstrating a 50% decrease in clonogenicity at a concentration of 4 mM 2DG. Variability to 2DG induced cytotoxicity has been previously reported and related to a multi-drug resistant phenotype (Kaplan *et al*, 1991). However, variability of 2DG induced cytotoxicity may be related to the complexity of uptake and processing or to the levels of pro-apoptotic or anti-apoptotic proteins (Amundson *et al*, 2000). We have further demonstrated that 2DG causes cell death rather than withdrawal from the cell cycle using clonogenic survival. The mechanism of cell death induced by 2DG appears to be by apoptosis as demonstrated by capase 3 and PARP activation. Slightly higher concentrations of 2DG were required to observe a significant amount of apoptosis compared with clonogenic survival in SkBr3 cells. This would be consistent with 2DG causing cell death by methods other than apoptosis exclusively such as necrosis (Sperandio *et al*, 2000; Tannock and Lee, 2001). Glucose deprivation results in increased glucose uptake and Glut1 transporter expression (Fisher and Frost, 1996). Under these conditions, an incompletely glycosylated form of Glut1 is synthesised which migrates as a 37 kDa protein (Reed *et al*, 1990; Kitzman *et al*, 1993; Macmahon *et al*, 2000). Our experiments demonstrate a similar response upon treatment of breast cancer cells with 2DG. 2DG treatment of breast cancer cells resulted in an increased glucose uptake as well as expression of higher levels of Glut1 glucose transporter. A lower molecular weight Glut1 protein was detected which likely represents an incomplete glycosylated form. From our results, we hypothesise that breast cancer cells treated with 2DG accelerate their own demise as a result of increased glucose transport as an initial response to 2DG.

Two properties of 2-DG, inhibition of glycolysis and preferential accumulation in cancer cells have formed the basis for further investigating the mechanism of 2DG for use as an anti-tumour agent. We speculate that cancer cells initially treated with 2DG exhibit a stress response due to a depletion of intracellular energy. The stress response results in increased levels of glucose transporter expression and increased glucose uptake, which allows more 2DG to enter the cell. As a consequence of high intracellular concentrations 2DG, hexokinase and hexose phosphate isomerase are inhibited, energy stores such as ATP are further depleted, and the cell activates the cell death pathway. Cancer cells are dependent on glycolysis for energy production and appear to be uniquely sensitive to glucose-deprivation and the effects of 2DG (Kaplan *et al*, 1991). The concentrations of 2DG which we observe to be cytotoxic to breast cancer cells *in vitro* are achievable *in vivo* (Mohanti *et al*, 1996). In several animal models of experimental malignancies tumour growth was inhibited with 2DG treatment (Laszlo *et al*, 1960; Kern and Norton, 1987; Ko *et al*, 2001). Clinical experience with 2DG has been favorable but limited (Landau *et al*, 1958; Mohanti *et al*, 1996). We think interference with glucose metabolism by tumour specific glucose analogues such as 2DG may serve as an effective agent or sensitiser to other therapeutic modalities especially in multidrug resistant cells.
